# Editorial: Incorporating non-thermal technologies to enhance food fortification strategies: current trends and future directions in personalized nutrition

**DOI:** 10.3389/fnut.2026.1878863

**Published:** 2026-06-08

**Authors:** Mustapha Muhammad Nasiru, Aliyu Idris Muhammad, Evans Frimpong Boateng

**Affiliations:** 1 College of Food Science and Engineering, Bohai University, Jinzhou, China; 2 Department of Agricultural and Environmental Engineering, Faculty of Engineering, Bayero University, Kano, Nigeria; 3 Department of Food Science and Technology, School of Agriculture and Technology, University of Energy and Natural Resources, Sunyani, Ghana

**Keywords:** food fortification, non-thermal technologies, nutrient delivery, nutrigenomics, personalized nutrition

Food fortification remains one of the most cost-effective public health strategies for addressing micronutrient deficiencies worldwide. However, traditional thermal processing often compromises heat-sensitive nutrients, reducing bioavailability and limiting efficacy in complex food matrices. Non-thermal technologies, such as high-pressure processing (HPP), pulsed electric fields (PEF), ultrasound, cold plasma, and thermosonication, offer powerful alternatives. These methods preserve or even enhance nutrient stability, improve microbial safety with minimal heat, and enable the better integration of fortificants, all while maintaining sensory and functional qualities (1).

This Research Topic explores the synergistic potential of non-thermal technologies with advanced fortification approaches, particularly in the emerging era of personalized nutrition. By minimizing nutrient degradation, facilitating targeted delivery through encapsulation or matrix modification, and supporting precision strategies informed by omics data, these innovations move beyond one-size-fits-all solutions toward foods tailored to individual genetic, metabolic, and lifestyle needs. The Research Topic highlights current applications, optimization techniques, novel fortificants, and forward-looking integrations that address sustainability, consumer acceptance, and health outcomes.

Contributions to this Research Topic demonstrate practical advancements across diverse matrices and technologies. For instance, Habeych et al. proposed using ferrous picolinate as a novel fortificant particularly suited for sensitive dairy products because traditional iron compounds often cause sensory or stability issues. Their study underscores how the careful selection of fortificant forms, potentially combined with non-thermal stabilization, can expand fortification options in challenging foods.

Alsanie conducted a comprehensive review integrating multi-omics approaches (genomics, proteomics, metabolomics, and microbiomics) with non-thermal processing technologies, including HPP, PEF, cold plasma, ultrasound, and supercritical fluid extraction. This review outlines a pathway to precision nutrition, wherein nutrient bioavailability is optimized based on individual profiles and supported by AI-driven modeling and advanced delivery systems.

Hardinsyah et al. provided a blueprint for vitamin D fortification in sugar, evaluating its stability and health impacts. The authors also offered a practical example of fortifying staple or widely consumed vehicles with attention to processing compatibility.

(Yikmiş, Altan, Demirel, et al.) contributed original research on the thermosonication-assisted fortification of kiwi juice with bee bread using an adaptive neuro-fuzzy inference system and response surface methodology (ANFIS-RSM) to optimize the process and enhance nutritional and functional properties. A related study on dill juice employed an advanced low-thermal fortification approach coupled with multilayer perceptron models (MLP-RSM) to improve bioaccessibility and functionality, illustrating how hybrid non-thermal or low-thermal methods, combined with modeling, can be tailored to specific juices and bioactives (Yikmiç, Altan, Türkol, et al.).

Collectively, these articles illustrate several key themes:

(1) Superior retention of thermolabile vitamins, minerals, and bioactives compared to conventional methods;
(2) Improved bioavailability through matrix disruption or encapsulation without excessive thermal load;
(3) Optimization via response surface methodologies, artificial intelligence, or ANFIS for process efficiency; and
(4) The bridge to personalization, in which omics insights guide the design of nutrient delivery matched to consumer needs.

In a broader context, these advances align with global challenges, including hidden hunger, malnutrition, the rising burden of chronic diseases, and the demand for sustainable, minimally processed foods. Non-thermal technologies generally consume less energy than prolonged thermal treatments and support cleaner-label products, although scalability, regulatory harmonization, and cost-effectiveness require continued attention. Integration of these technologies with personalized nutrition, leveraging nutrigenomics and real-time biomarkers, promises more equitable and effective interventions, shifting the focus from population-level fortification to individualized functional foods.

Future directions highlighted across this Research Topic include the deeper convergence of non-thermal processing with biotechnology (e.g., precision fermentation), smart delivery systems, life-cycle assessments for sustainability, and rigorous clinical validation of health impacts. Sensory profiling and consumer studies will remain essential to ensure market success. As the field matures, interdisciplinary collaboration among food technologists, nutritionists, data scientists, and policymakers will be critical to translate these innovations into accessible solutions that enhance public health.

This Research Topic provides a timely insight into innovations at the intersection of food processing, fortification, and personalization. It lays the groundwork for next-generation strategies that preserve nutritional integrity while respecting individual variability, ultimately supporting more resilient and health-promoting food systems.
